# Supplementary material for: Transcriptome Analysis of Differentially Expressed mRNA Related to Pigeon Muscle Development
Source: Animals (Basel). 2021 Aug 5;11(8):2311. doi: 10.3390/ani11082311 (PMC8388485; doi:10.3390/ani11082311)
Supplement: Supplementary file 1 [file animals-11-02311-s001.zip › Table S1 and Table S2.pdf]

Supplementary Materials: Table S1. Statistics of the number of transcripts detected by each sample. Table S2. Statistical table of mRNA transcript results of inter group differences.

Table S1. Statistics of the number of transcripts detected by each sample.

Sample Name	Known Isoform Num	New Isoform Num	All Isoform Num
E8-1	13767 (75.86%)	11923	25690
E8-2	13953 (76.88%)	12189	26142
E8-3	13772 (75.89%)	11965	25737
E13-1	13355 (73.59%)	11883	25238
E13-2	13580 (74.83%)	12009	25589
E13-3	13487 (74.32%)	11895	25382
D1-1	13325 (73.42%)	11699	25024
D1-2	13119 (72.29%)	11546	24665
D1-3	13214 (72.81%)	11604	24818
D10-1	13410 (73.89%)	11615	25025
D10-2	13511 (74.45%)	11579	25090
D10-3	13280 (73.18%)	11617	24897

Table S2. Statistical table of mRNA transcript results of inter group differences

Pairs	DiffGene(Up)	DiffGene(Down)	All DiffGene
D1-vs-D10	1019	1341	2360
E13-vs-D1	1095	1557	2652
E13-vs-D10	1776	3182	4958
E8-vs-D1	1940	4084	6024
E8-vs-D10	2368	5874	8242
E8-vs-E13	902	1057	1959
